# Benzoic Acid, Chlorine Dioxide, and 1-Methylcyclopropene Induce Flavonoid Metabolic Shifts in Postharvest Flowering Chinese Cabbage Revealed by High-Dimensional Analytical Data

**DOI:** 10.3390/ijms23116011

**Published:** 2022-05-27

**Authors:** Lingqi Yue, Yongshen Li, Min Zhong, Xirong Chai, Puyan Zhao, Riming Huang, Yunyan Kang, Xian Yang

**Affiliations:** 1 College of Horticulture, South China Agricultural University, Guangzhou 510642, China; ylq20211017004@stu.scau.edu.cn (L.Y.); lysscau@163.com (Y.L.); zhongmin@scau.edu.cn (M.Z.); chaixirong1006@163.com (X.C.); zhaopuyan@scau.edu.cn (P.Z.); 2 Guangdong Provincial Key Laboratory of Food Quality and Safety, College of Food Science, South China Agricultural University, Guangzhou 510642, China; huangriming@scau.edu.cn

**Keywords:** *Brassica campestris* L. ssp. *chinensis* var. *utilis* Tsen et Lee, benzoic acid, chlorine dioxide, 1-methylcyclopropene, flavonoid, phenylpropanoid pathway

## Abstract

Flowering Chinese cabbage (*Brassica campestris* L. ssp. *chinensis* var. *utilis* Tsen et Lee) is one of the most popular vegetables in China. However, the loss of the functional ingredients in postharvest flowering Chinese cabbage during storage is still serious, owing to the unclear causes of the metabolic shifts. Herein, benzoic acid, chlorine dioxide, and 1-methylcyclopropene (1-MCP) could maintain the quality of postharvest flowering Chinese cabbage, and 1-MCP showed the best effect. Furthermore, transcript-metabolite profiling of the treatments revealed a transcript-metabolite correlation network of the flavonoid biosynthesis pathways with a range of 3 to 3662 differentially expressed genes (DEGs) and a range of 23 to 37 differentially accumulated metabolites (DAMs). Surprisingly, 1-MCP had the best effect on shelf life among the treatments, although chlorine dioxide could stimulate the expression of four critical differential genes (*Bra007142*, *Bra008792*, *Bra009358*, and *Bra027457*) involved in delaying flavonoid degradation (hesperetin, chalcone, rutin, baicalein). As a result, our findings will help to improve our understanding of the regulation of flavonoid production in relation to the quality of postharvest flowering Chinese cabbage during storage.

## 1. Introduction

Preservation technology is critical for vegetables because of their perishable nature [1]. Chemical preservatives, in comparison to other preservation strategies, play an exceptionally important role in postharvest vegetables due to their ease of application and low cost [2]. With the advancement of living standards, the functional elements of vegetables have become increasingly important [3]. As a result, the alteration of functional components in postharvest vegetables during storage should be a major concern [4].

Flowering Chinese cabbage is a popular cruciferous vegetable that is commercially distributed in China [5]. Flowering Chinese cabbage is very popular because it is rich in several nutrients and active phytochemicals, e.g., minerals, dietary fiber, vitamins C and E, glucosinolates, phenolic acids, carotenoids, flavonoids, and anthocyanins, which can be beneficial to human health [6,7]. The previous investigations mainly focused on the physiological-biochemical characteristics of flowering Chinese cabbage during the preharvest stage [8,9]. However, the loss of the functional ingredients in postharvest flowering Chinese cabbage during storage is ignored. Furthermore, the causes of differential metabolism in postharvest flowering Chinese cabbage treated with chemical substances with preservative effects during storage are still unclear [10]. Thus, great efforts are urgently needed to evaluate the specific causes of differential metabolism in the quality of postharvest flowering Chinese cabbage during storage.

Benzoic acid, chlorine dioxide, and 1-methylcyclopropene (1-MCP) are commonly used to prolong the shelf life of postharvest fruit and vegetables [11,12,13]. It is reported that preharvest application of benzoic acid can increase the content of total phenolic compounds and total anthocyanins in table grapes [14], and postharvest antimicrobial treatments with benzoic acid can improve the shelf life of fresh blueberries [15]. Chlorine dioxide can control postharvest anthracnose disease in mango [16], and delays the reddening of postharvest green peppers by affecting the chlorophyll degradation and carotenoid synthesis pathways [17]. Guo et al. (2014) indicate that chlorine dioxide treatment might delay the ripening of tomato fruit, possibly by a mechanism involving suppression of respiration rate and ethylene biosynthesis [18]. 1-MCP has been widely used to delay senescence and maintain postharvest quality in vegetables [19,20,21,22,23,24,25]. Current studies on broccoli and pepper showed that 1-MCP can retard chlorophyll degradation, reducing the expression of chlorophyll degradation associated genes [20,21,22,24]. Additionally, Chlorine dioxide and 1-MCP can regulate the activities of L-phenylalanine ammonia-lyase (PAL), superoxide dismutase, and catalase, which results in increasing the content of total phenols and flavonoids in crops [26,27]. Given the synergistic inhibitory effect of 1-MCP and chlorine dioxide treatments on chlorophyll degradation of green pepper fruit during storage [28], the studies of the comparative effects of benzoic acid, chlorine dioxide, and 1-MCP on the preservation and flavonoid metabolism of vegetables and fruit are limited, and the scientific effects of these three chemical substances on postharvest flowering Chinese cabbage have not been explored.

Plant-derived secondary metabolites exhibit enormous structural variation and a diversity of biological properties [29]. In recent years, lots of research has focused on secondary metabolites because of their potential as health-promoting phytochemicals [30]. The growing evidence of functional flavonoids’ benefits to human health has driven consumers to demand functional flavonoid-rich food products [31]. Furthermore, flavonoids have a great number of functions in Brassicaceae vegetables [32]. For example, the colorless flavonoids accumulated in the outermost layers of plants can absorb UV radiation to prevent harmful effects on the internal tissues [33]. Although the bioactive flavonoids of some vegetables have been investigated [3], they are highly influenced by geographical region, cultivar, water availability, climate, light exposure, degree of ripeness, and storage conditions [31,33,34]. The most common flavonoids in Brassica crops are quercetin, isorhamnetin, and kaempferol, mainly conjugated to glucose, commonly found as O-glycosides. They are also commonly acylated by different hydroxycinnamic acids [35]. It is important to consider that not all vegetables have the same flavonoid components, and not all flavonoids possess the same functional properties. Thus, it is necessary to clarify the key genes and their regulated flavonoids that are annotated in the biosynthesis pathways of flavonoids with benefit to the quality of postharvest flowering Chinese cabbage treated with chemical substances with preservative effects.

In our present study, based on investigating the effects of benzoic acid, chlorine dioxide, and 1-MCP on prolonging the shelf life of postharvest flowering Chinese cabbage, we focused on the flavonoid metabolic shifts and molecular mechanism, and found out which chemical substances could both prolong the shelf life and delay the degradation of flavonoids. These molecular details of benzoic acid, chlorine dioxide, and 1-MCP on the postharvest flowering Chinese cabbage during storage were revealed by high-dimensional analytical data, including transcript-metabolite profiling, reverse transcription quantitative real-time polymerase chain reaction (qRT-PCR), and high-performance liquid chromatography (HPLC) analysis.

## 2. Results and Discussion

### 2.1. The Phenotype and Quality of Postharvest Flowering Chinese Cabbage Treated with Chlorine Dioxide, Benzoic Acid, and 1-MCP

**Figure 1:**
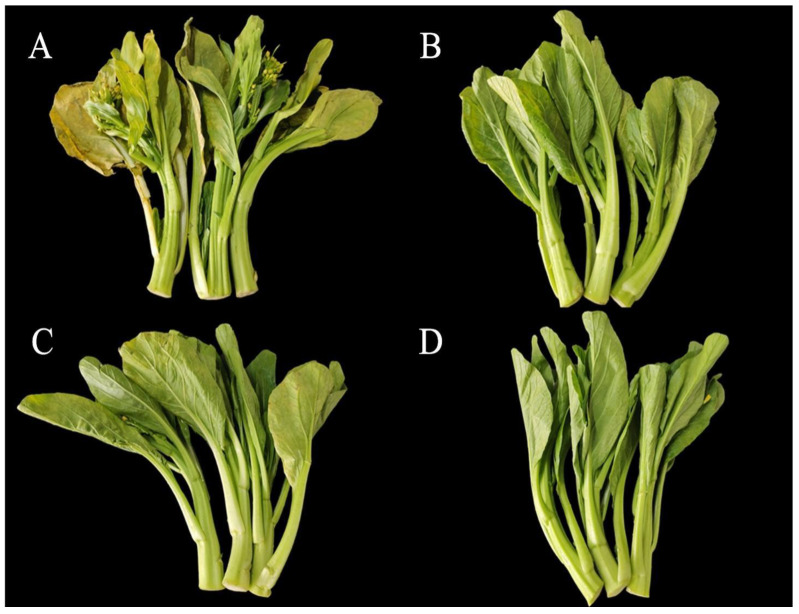
The phenotype of postharvest flowering Chinese cabbage treated with benzoic acid, chlorine dioxide, and 1-MCP was stored at 4 °C for 20 days. (**A**) control group, (**B**) chlorine dioxide treated group, (**C**) benzoic acid treated group, and (**D**) 1-MCP treated group.

**Figure 2:**
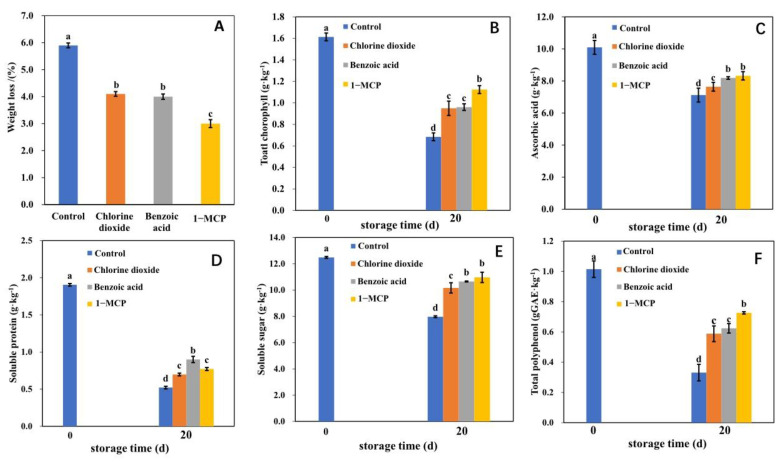
The weight loss (**A**), the concentration of chlorophyll (**B**), ascorbic acid (**C**), soluble protein (**D**), soluble sugar (**E**), and total polyphenol (**F**) of postharvest flowering Chinese cabbage treated with benzoic acid, chlorine dioxide, and 1-MCP. The *x*-axis represents the storage time. The vertical bars show the standard error of the mean (*n* = 3). The different letters indicate statistically significant differences for each group (*p*  <  0.05).

Cefola et al. (2010) suggested that 1-MCP extended the shelf life of broccoli, reducing postharvest deterioration, retarding chlorophyll degradation, and delaying visual quality loss [36]. Recently, chlorine dioxide has been utilized for the postharvest green peppers to delay the reddening by affecting the chlorophyll degradation and carotenoid synthesis pathways [17]. Yao et al. (2020) reported that benzoic acid could prolong the shelf life of flowers, however, the effects on postharvest physiology remained scarce [37]. It was reported that the regulatory roles between 1-MCP and chlorine dioxide in delaying aging were different [28]. Flowering Chinese cabbage is a kind of leaf vegetable, and the leaf yellowing is a typical characteristic of plant senescence. As is shown in Figure 1, there were visible differences in the degree of yellowing, wilting, and water loss among the treatments after 20 d of storage. The leaves of the control group displayed the most serious yellowing phenomenon. The leaves of chlorine dioxide and benzoic acid treated groups showed a certain extent of yellowing, leaf edge crimping, and water loss. However, the leaves treated with 1-MCP were green and shiny, without obvious yellowing and water loss. The analysis of the physiological qualities indicated that the three treatments demonstrated different effects on weight loss, and the concentration of chlorophyll, ascorbic acid, soluble protein, soluble sugar, and polyphenol in postharvest flowering Chinese cabbage after 20 d of storage (Figure 2). As shown in (Figure 2A), we found that the weight loss of 1-MCP, benzoic acid, and chlorine dioxide treated groups, and the control group decreased by 3.0%, 4.0%, 4.1%, and 5.9%, respectively. The maximum chlorophyll concentration was observed under the 1-MCP treatment, while the minimum chlorophyll concentration was recorded under the control (Figure 2B). However, there was no significant difference in weight loss and chlorophyll concentration between benzoic acid and chlorine dioxide treatments. These findings indicated that 1-MCP had the best effect on controlling the senescence of leaves, followed by benzoic acid and chlorine dioxide. This is consistent with the result obtained in plant phenotype.

Previous reports have shown that there are differences in the effects on the postharvest quality of different crops after storage between chlorine dioxide and 1-MCP [28,38]. In this study, the ascorbic acid concentration of 1-MCP, benzoic acid, chlorine dioxide treated, and the control group decreased by 16.8%, 18.1%, 24.4%, and 29.5%, respectively (Figure 2C). The results showed that the ascorbic acid concentration was degraded in the treatments, but the degradation of the three treatments was slower than that of the control, and the best effect was observed in the 1-MCP treatment. The soluble protein concentration of 1-MCP, benzoic acid, chlorine dioxide treated, and the control groups decreased by 64.1%, 52.8%, 66.4%, and 72.3%, respectively (Figure 2D), which indicated benzoic acid had the best effect on controlling the loss of soluble protein. The soluble sugar concentration of 1-MCP, benzoic acid, and chlorine dioxide treated groups and the control group decreased by 12.2%, 14.7%, 18.6%, and 36.1%, respectively (Figure 2E). The results demonstrated that 1-MCP could better delay the degradation of soluble sugar, but there was no significant difference between 1-MCP and benzoic acid treatments. Similarly, the treatments could reduce the total polyphenol concentration after 20 d of storage. The polyphenol concentration of 1-MCP, benzoic acid, and chlorine dioxide treated groups and the control group decreased by 25.9%, 36.4%, 41.6%, and 66.3%, respectively (Figure 2F).

The above differences of the treatments may be related to their different mechanisms. 1-MCP and chlorine dioxide delayed senescence and maintained postharvest quality by inhibiting chlorophyll degradation, ethylene production, and respiration, as well as repressing the expression of chlorophyll degradation and ethylene synthesis genes [17,20,28,38]. However, 1-MCP and chlorine dioxide played different regulation roles in the chlorophyll degradation pathway as they had different inhibitory effects on *CaCLH* expression, and the effects on suppressing the expression of genes involved in the chlorophyll breakdown pathway were ranked as follows: 1-MCP > chlorine dioxide [28]. In addition, chlorine dioxide could maintain postharvest quality by inhibiting the occurrence of pathogens, but 1-MCP did not [38]. Currently, available data on the mechanisms of delaying senescence in fruits and vegetables under the influence of benzoic acid is limited. Only a few studies mention that the use of benzoic acid could inhibit the occurrence of pathogens [39]. Compared with the control, 1-MCP, benzoic acid, and chlorine dioxide could delay the senescence and nutrient degradation of postharvest flowering Chinese cabbage, while 1-MCP treatment showed the strongest positive influence. We speculate that the main reason for the best performance shown by 1-MCP treatment is perhaps that it can more effectively inhibit respiration, ethylene production, and chlorophyll degradation, and suppress the expression of the genes associated with chlorophyll degradation and ethylene synthesis.

### 2.2. Transcript-Metabolite Profiling of Postharvest Flowering Chinese Cabbage Treated with Chlorine Dioxide, Benzoic Acid, and 1-MCP

#### 2.2.1. Transcriptome Analysis

To analyze the level of transcription, RNA-seq was carried out using equal amounts of RNA from the postharvest flowering Chinese cabbage treated with benzoic acid, chlorine dioxide, and 1-MCP (Figure 1).

**Figure 3:**
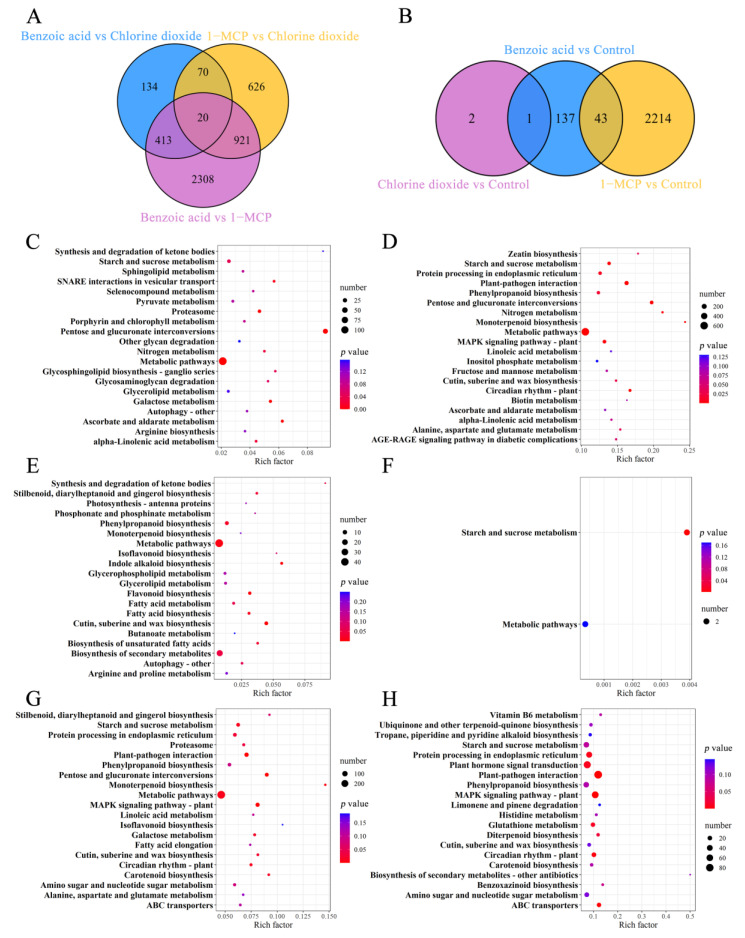
Venn diagram revealing DEGs numbers from different groups (**A**,**B**). The numbers of DEGs and significantly enriched KEGG pathways in different combinations of Benzoic acid vs. Chlorine dioxide (**C**), Benzoic acid vs. 1-MCP (**D**), Benzoic acid vs. Control (**E**), Chlorine dioxide vs. Control (**F**), 1-MCP vs. Chlorine dioxide (**G**), 1-MCP vs. Control (**H**). The *y*-axis and *x*-axis are KEGG pathways and enrich factors, respectively. Low and high *p* values are expressed by the red circle and the blue circle, respectively. The area of a circle is the DEG number.

Comparisons between the differentially expressed genes (DEGs) of the control group and the three treatment groups (20 d of storage) were illustrated in Figure 3A,B. The numbers of DEGs ranged from 3662 of “Benzoic acid vs. 1-MCP” to 3 of “Chlorine dioxide vs. Control”. Three compared combinations, “1-MCP vs. Chlorine dioxide” and “Benzoic acid vs. Chlorine dioxide”, “Chlorine dioxide vs. Control” and “1-MCP vs. Control”, and “1-MCP vs. Control” and “Benzoic acid vs. Control”, shared 90, 1, and 43 DEGs, respectively, which revealed that these different common DEGs continued to play an important function during the entire 20 d storage. Therefore, this DEG profile emphasized the very active gene expression in withered leaves of postharvest flowering Chinese cabbage, and that the dehydration stress might produce transcriptional changes during the storage.

The relationship between DEGs and their related biosynthesis pathways was performed by searching the Kyoto Encyclopedia of Genes and Genomes (KEGG) pathway database according to these DEGs to reveal the top 20 significantly enriched pathways (Figure 3C–H). Pathways of “metabolic pathway” in “1-MCP vs. Chlorine dioxide”, “Benzoic acid vs. 1-MCP” and “Benzoic acid vs. Chlorine dioxide” groups, “plant-pathogen interaction” and “MAPK signaling pathway” in “1-MCP vs. Control” groups, “biosynthesis of secondary metabolites” in “Benzoic acid vs. Control” group, “starch and sucrose metabolism” in “Chlorine dioxide vs. Control” group were significantly active. The DEGs of three treatments were significantly enriched in these pathways, as mentioned above. Additionally, pathways linked to the accumulation of “metabolic pathways” significantly enriched in these groups. These transcriptome data indicated that dramatic changes in gene expression would result in an alteration of the contents and proportion of some functional constituents, such as flavonoids, amino acids, volatiles, and soluble sugars.

#### 2.2.2. Metabolite Analysis

**Figure 4:**
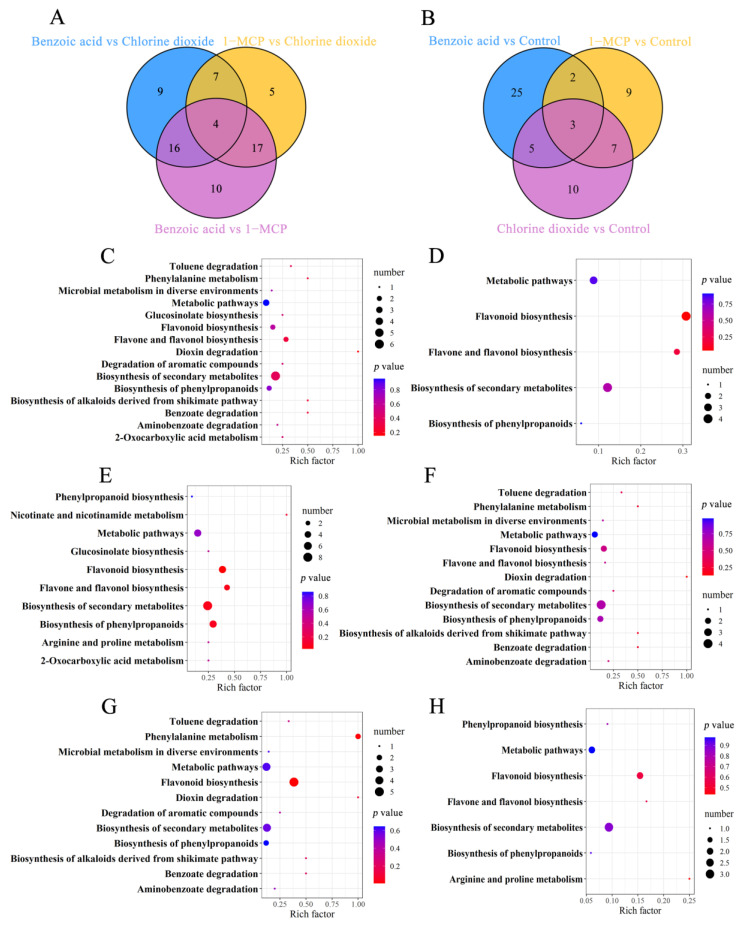
Venn diagram revealing DAMs numbers from different combinations (**A**,**B**). KEGG pathway enrichment analysis of DAMs in different combinations of Benzoic acid vs. Chlorine dioxide (**C**), Benzoic acid vs. 1-MCP (**D**), Benzoic acid vs. Control (**E**), Chlorine dioxide vs. Control (**F**), 1-MCP vs. Chlorine dioxide (**G**), 1-MCP vs. Control (**H**). The *x*-axis and *y*-axis represent the enrichment factor and the enrichment pathway, respectively. Low and high *p* values are expressed by the red circle and the blue circle, respectively. The area of a circle is the DAM number. The enrichment was performed by Fisher’s exact test.

The metabolite profiles of postharvest flowering Chinese cabbage treated with benzoic acid, chlorine dioxide, and 1-MCP were detected by the essential spectrum of quality control samples (Figure S1) and then subjected to the principal component analysis (PCA). The PCA score plots showed an apparent separation resolution between the two groups (Figure S2). The results of the orthogonal partial least square-discriminant analysis (OPLS-DA) revealed that the dominating biological metabolites were changed between the two groups (Figures S3–S5). The differentially accumulated metabolites (DAMs) with a fold change ≥2 or a fold change ≤0.5 were considered of variable importance in the project (VIP) ≥1 between the control and each treatment of chlorine dioxide, benzoic acid, and 1-MCP (Figures S6 and S7, and Table S2). The numbers of DAMs ranged from 37 of “Benzoic acid vs. 1-MCP” to 23 of “1-MCP vs. Chlorine dioxide”. Three compared combinations, “Benzoic acid vs. Chlorine dioxide” and “1-MCP vs. Chlorine dioxide”, “Benzoic acid vs. Control” and “1-MCP vs. Chlorine dioxide”, and “1-MCP vs. Control” and “Chlorine dioxide vs. Control”, shared 11, 5, and 10 DAMs, respectively, which suggested that these differential DAMs continued to play a function during the entire 20 d storage. DAMs in the benzoic acid, chlorine dioxide, and 1-MCP treatments vs. the control were functionally annotated based on the KEGG database. The KEGG enrichment analysis revealed that the terms “flavonoid biosynthesis”, “biosynthesis of phenylpropanoids”, “biosynthesis of secondary metabolites” in “Benzoic acid vs. Control’, “flavonoid biosynthesis”, “biosynthesis of secondary metabolites”, “flavone and flavonol biosynthesis” in “Benzoic acid vs. 1-MCP”, “biosynthesis of secondary metabolites” in “Chlorine dioxide vs. Control”, “flavonoid biosynthesis”, “biosynthesis of secondary metabolites”, “biosynthesis of phenylpropanoids” in “Benzoic acid vs. 1-MCP”, “biosynthesis of secondary metabolites” in “1-MCP vs. Control”, “biosynthesis of phenylpropanoids” in “Benzoic acid vs. Chlorine dioxide”, were significantly enriched (Figure 4). The DAMs of benzoic acid, chlorine dioxide, and 1-MCP treatments were significantly enriched in these pathways mentioned above. These data indicated that chlorine dioxide, benzoic acid, and 1-MCP had different effects on the contents and proportion of flavonoids in postharvest flowering Chinese cabbage.

#### 2.2.3. Transcript-Metabolite Correlation Network

**Figure 5:**
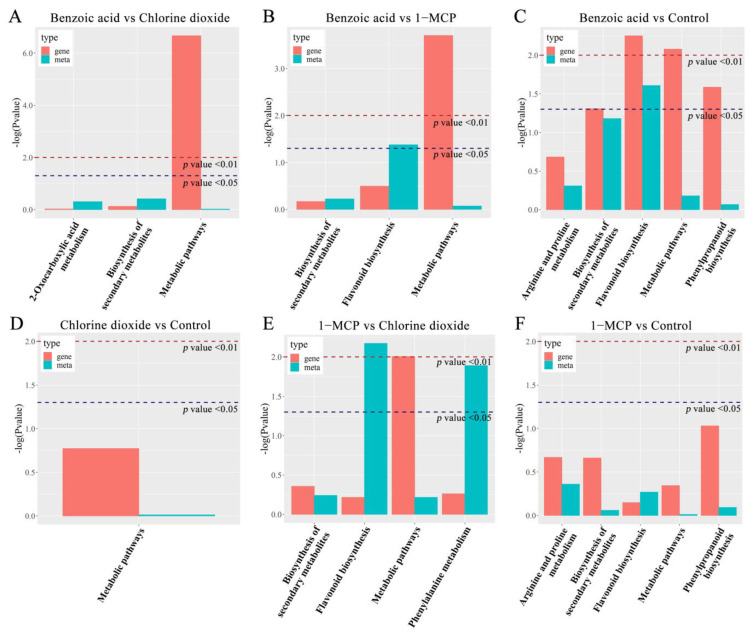
KEGG pathway enrichment analysis of DAMs and DEGs in different combinations of Benzoic acid vs. Chlorine dioxide (**A**), Benzoic acid vs. 1-MCP (**B**), Benzoic acid vs. Control (**C**), Chlorine dioxide vs. Control (**D**), 1-MCP vs. Chlorine dioxide (**E**), 1-MCP vs. Control (**F**). The *x*-axis is the enrichment pathway, while red and green on the *y*-axis are the *p*-values of DAMs and DEGs, respectively.

To analyze the change of the DAMs and DEGs in the postharvest flowering Chinese cabbage induced by chlorine dioxide, benzoic acid, and 1-MCP, the network was established to confirm transcript-metabolite correlations. In our present study, the correlation pairs related to the functionally annotated signaling pathways were included in the analysis (Figure 5). The visualized network revealed that the DAMs were connected by corresponding DEGs (Figure 5). In the benzoic acid, chlorine dioxide, and 1-MCP treatments vs. the control, four differential metabolites were involved in the transcript-metabolite co-mapped correlation network of flavonoid biosynthesis pathways. It indicated that benzoic acid, chlorine dioxide, and 1-MCP might affect the metabolism of flavonoids in postharvest flowering Chinese cabbage through another new signaling pathway, and also give new insight into the quality-related components of postharvest flowering Chinese cabbage during storage [40].

The analysis of widely targeted metabolite data using MS/MS profiling has previously been applied for large-scale metabolite data and comparative metabolomics of many important plant species [41]. Previous reports mainly focused on the primary constituents of flowering Chinese cabbage, such as chlorophyll (2.803 g kg^−1^), soluble sugar (2.0%), and soluble protein (5.9%) [8]. However, to date, over-arching differences in the functional metabolic shifts of flowering Chinese cabbage have not been thoroughly investigated yet. In our present study, we utilized LC-MS/MS-based widely targeted genes and metabolomics to understand better the metabolite variations of chlorine dioxide, benzoic acid, and 1-MCP treatments. In the transcript-metabolite analysis, the numbers of DEGs ranged from 3662 of “Benzoic acid vs. 1-MCP” to 3 of “Chlorine dioxide vs. Control”. The numbers of DAMs ranged from 37 of “Benzoic acid vs. 1-MCP” to 23 of “1-MCP vs. Chlorine dioxide”, which revealed that these differential DEGs and DAMs continued to play a function during the entire 20 d storage. The transcript-metabolite profiling demonstrated that benzoic acid, chlorine dioxide, and 1-MCP co-induced three differentially enriched metabolites, which were baicalein [42], nicotiflorin [43], and 4-methylsulfonyl-3-butenyl glucosinolate [44], functional components in many edible plants [45,46]. Furthermore, the transcript-metabolite co-mapped the correlation network of flavonoid biosynthesis pathways that plays an important role in the formation of functional components in vegetables [47,48], which indicated that benzoic acid, chlorine dioxide, and 1-MCP could affect the metabolism of flavonoids in flowering Chinese cabbage through the flavonoid biosynthesis pathway [49]. To investigate the functional differential genes and their regulating flavonoids, the transcript-metabolite analysis revealed that there were 127 DEGs and 9 differential flavonoid metabolites rich in the flavonoid biosynthetic pathway. The 9 differential flavonoids were kaempferol-3-O-rutinoside (nicotiflorin) (PubChem CID:5318767), eriodictyol, baicalein (PubChem CID:5281605), catechin (PubChem CID:9064), chalcone (PubChem CID:637760), hesperetin (PubChem CID:72281), taxifolin (PubChem CID:439533), kaempferol, and rutin (PubChem CID:5280805). Nicotiflorin, eriodictyol, baicalein, catechin, chalcone, hesperetin, taxifolin; kaempferol, and rutin are rich in flavonoid biosynthesis paths. Moreover, the metabolomic results displayed that most of the flavonoids in the differential metabolites were in the form of flavonoid glycosides. In the flavanone biosynthesis pathway, p-coumaroyl-CoA forms naringenin chalcone through the *Bra008792* gene, and then naringenin and hesperetin through the *Bra007142* gene. Hesperetin forms hesperetin 7-O-glucoside through flavanone 7-O-β-glucosyl transferase. Furthermore, neohesperidin was formed through flavanone 7-O-glucoside 2-O-β-L-rhamnoside transferase. Besides, taxifolin regulated the *Bra009358* gene in combination with flavanoid 3′,5′-hydroxylase to form quercetin, then formed isoquercitrin through flavonol 3-O-glucosyl transferase. Isoquercitrin could then form quercetin 3-O-(6-O-malonyl-β-D-glucoside), quercetin-3-O-β-D-xylopyranoside, quercetin-3-O-rutinoside (rutin), quercetin 3-O-beta-D-glucosyl-(1->2)-β-D-glucoside and other substances through various transferases. However, these functional differential genes and their regulating flavonoids metabolites should be further confirmed.

### 2.3. The Confirmation of Functional Differential Genes and Their Regulating Flavonoids Metabolites

**Figure 6:**
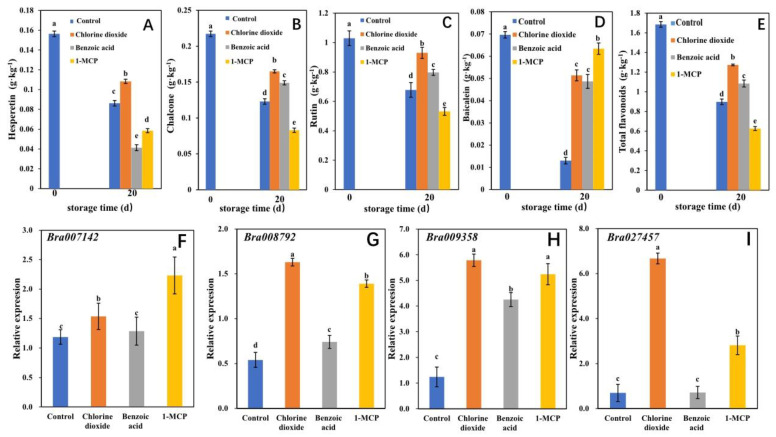
The concentrations of hesperetin (**A**), chalcone (**B**), rutin (**C**), baicalein (**D**), and total flavonoids (**E**) in postharvest flowering Chinese cabbage treated with benzoic acid, chlorine dioxide, and 1-MCP after storage. The gene expression of *Bra007142* (**F**), *Bra008792* (**G**), *Bra009358* (**H**), and *Bra027457* (**I**) in flowering Chinese cabbage treated with benzoic acid, chlorine dioxide, and 1-MCP. The vertical bars show the standard error of the mean (*n* = 3). The different letters indicate statistically significant differences for each group (*p*  <  0.05).

Based on the analysis of the transcript-metabolite correlation network, the genes regulating the differential flavonoids in chlorine dioxide, benzoic acid, and 1-MCP treatments with large multiply differences were *Bra007142*, *Bra008792*, *Bra009358*, and *Bra027457* genes. These genes regulated the flavonoids of hesperetin, chalcone, quercetin-3-O-rutinoside, and baicalein, respectively. The concentration of hesperetin, chalcone, rutin, baicalein, and total flavonoids after 0 d and 20 d of storage were quantitated and quantified by HPLC compared with their standard references (Figure 6A–E). Furthermore, the four differential genes were confirmed by qRT-PCR technology and their regulating differential flavonoids were quantified (Figure 6F–I).

Figure 6A–E showed that the concentration of rutin in postharvest flowering Chinese cabbage was the highest (1.030 g kg^−1^) at 0 d, followed by chalcone (0.217 g kg^−1^), then hesperetin (0.156 g kg^−1^) and the concentration of baicalein was the lowest (0.070 g kg^−1^). After 20 d of storage, the concentration of rutin, chalcone, hesperetin, baicalein, and total flavonoids decreased by 34.1%, 43.4%, 44.9%, 81.3%, and 46.7%, respectively. Our present results agreed with the findings from the study of hardy kiwifruit by Jeong et al. (2020) [50], where it could be seen that cv. Chiak showed a significant decrease in total flavonoid content at 8 weeks of storage. The decrease of flavonoid content is related to the decrease of PAL content during storage [51].

During storage, the concentration of the total flavonoids and four flavonoids including hesperetin, chalcone, rutin, and baicalein were all affected by benzoic acid, chlorine dioxide, and 1-MCP. However, the effects of the three treatments on each kind of flavonoid and the same treatment on different flavonoids were not consistent. It is reported that the application of benzoic acid [14], chlorine dioxide [38] and 1-MCP [52] could increase the total flavonoids contents in some crops. After 20 d of storage, the concentration of hesperetin in the chlorine dioxide treatment was higher than that in the control, while the concentration of hesperetin in benzoic acid and 1-MCP treatments was lower than that in the control, and the concentration of hesperetin in the benzoic acid treatment was the lowest. These findings indicated that both 1-MCP and benzoic acid treatment could promote the degradation of hesperetin while chlorine dioxide treatment delayed the degradation of hesperetin. After 20 d of storage, the concentration of chalcone and rutin in chlorine dioxide, benzoic acid, and 1-MCP treatments decreased successively and displayed differences. The concentration of chalcone and rutin of chlorine dioxide and benzoic acid treatments was higher than those in the control, which indicated that chlorine dioxide and benzoic acid could delay the degradation of chalcone and rutin, while the degradation delayed in the chlorine dioxide treatment was the best. The 1-MCP treatment promoted the degradation of chalcone and rutin, resulting in the concentration of chalcone and rutin in the 1-MCP treatment being lower than that in the control. The baicalein concentration of the three treatments was higher than that of the control, and the 1-MCP treatment was the highest. The results indicated that benzoic acid, chlorine dioxide, and 1-MCP could delay the degradation of baicalein and the 1-MCP showed the best effect. Benzoic acid, chlorine dioxide, and 1-MCP also showed different effects on the total flavone concentration of postharvest flowering Chinese cabbage. The total flavone concentration in the chlorine dioxide treatment was the highest, followed by the benzoic acid treatment, then the 1-MCP treatment. After 20 d storage of the control, chlorine dioxide, benzoic acid, and 1-MCP treatments, the total flavonoid concentration in postharvest flowering Chinese cabbage decreased by 49.7%, 24.4%, 35.7%, and 62.8%, respectively. The results indicated that chlorine dioxide could delay the degradation of flavone, while 1-MCP could promote flavonoid degradation. This finding was in agreement with other reports showing that treatment with chlorine dioxide enhanced the postharvest synthesis of flavonoids and nonflavonoids, an effect opposite to that of 1-MCP [38]. Chlorine dioxide might delay the degradation of flavonoids by increasing the activity of the PAL and promoting the metabolism of phenylpropane, while 1-MCP might present the opposite [38,52,53].

Flavonoid metabolism is a complex process, which is regulated by many genes. Some publications reported an increase in flavonoid content during senescence in grape berries [54] and apples [55] by an increase in flavonoid pathway gene expression. However, another behavior was described by Chaudhary et al. (2017), who reported a decrement in *PAL*, *CHS*, and *CHI* gene expression in grapefruit, and all four genes showed different expression patterns during storage [56]. Reyes Jara et al. suggested that *BoPAL* was more relevant than *BoCHS* and *BoCHI* in the biosynthesis of flavonoids during postharvest senescence of broccoli [57]. Moreover, Wu et al. (2018) observed an increase in *PAL* and *CHS*, and fewer changes in *CHI* expression under 1-MCP treatment in peach [58]. In this study, Figure 6F–I showed that *Bra007142*, *Bra008792*, *Bra009358*, and *Bra027457* presented different expression levels in the leaves of postharvest flowering Chinese cabbage. In the control group, the expression level of *Bra009358* was the highest, followed by *Bra007142* and *Bra027457*, then *Bra008792*. Benzoic acid, chlorine dioxide, and 1-MCP had different effects on the expression levels of *Bra007142*, *Bra008792*, *Bra009358*, and *Bra027457* in the leaves. The results showed that the *Bra007142* expression of 1-MCP, chlorine dioxide, and benzoic acid treatments increased by 111.8%, 45.9%, and 12.5%, respectively. Compared with the control, the *Bra007142* expression of chlorine dioxide and 1-MCP treatments showed a significant difference, while benzoic acid treatment displayed no significant difference. The *Bra008792* expression of chlorine dioxide, 1-MCP, and benzoic acid treatments decreased successively, and increased by 200.9%, 157.4%, and 37.0%, respectively, compared with the control. The variation trend of *Bra009358* expression was consistent with that of the *Bra008792*. The expression of the *Bra009358* in chlorine dioxide, 1-MCP, and benzoic acid treatments also decreased successively, but increased by 365.3%, 321.3%, and 241.9%, respectively, compared with the control. However, there was no significant difference between chlorine dioxide and 1-MCP treatments. The *Bra027457* expression of chlorine dioxide, 1-MCP, and benzoic acid treatments also decreased successively, and increased by 857.9%, 302.1%, and 2.9%, respectively, compared with the control. However, there was no significant difference between the benzoic acid treatment and the control, while the expression in chlorine dioxide and 1-MCP treatments was higher than that in the control. The above results showed that both chlorine dioxide and 1-MCP could induce the expression of *Bra007142*, *Bra008792*, *Bra009358*, and *Bra027457*. Benzoic acid could only increase the expression of *Bra009358* and *Bra008792*, while the expression of *Bra007142* and *Bra027457* was not significantly affected. It revealed that chlorine dioxide and 1-MCP could both positively regulate the expression of *Bra007142*, *Bra008792*, *Bra009358*, and *Bra027457*, while benzoic acid could only positively regulate the expression of *Bra009358* and *Bra008792* but could not show a regulation effect on the other two genes.

Interestingly, the regulation effects of chlorine dioxide, benzoic acid, and 1-MCP on the four genes in postharvest flowering Chinese cabbage showed differences, and different treatments could delay or promote the degradation of flavonoids by regulating the expression of different related genes. The chlorine dioxide treatment delayed the degradation of hesperetin, chalcone, rutin, and baicalein, as well as total flavonoids, for which both concentrations were higher than that in the control by up-regulating the expression of *Bra007142*, *Bra008792*, *Bra009358*, and *Bra027457*. 1-MCP could up-regulate the expression of *Bra007142*, *Bra008792*, *Bra009358*, and *Bra027457*. The synergistic regulation promoted the degradation of hesperetin, chalcone, rutin, and total flavone, while it delayed the degradation of baicalein, which resulted in the concentration of hesperetin, chalcone, rutin, and total flavonoids being lower than those of the control, but the content of baicalein was higher than that of the control. Benzoic acid delayed the degradation of chalcone, rutin, baicalein, and total flavone by up-regulating the expression of *Bra009358* and *Bra008792* genes. Compared with the control, the concentration of chalcone, rutin, baicalein, and total flavonoid in the benzoic acid treatment increased, while the concentration of hesperetin decreased. To date, the available data on the molecular mechanism of regulating flavonoid biosynthesis in postharvest vegetables under the influence of chlorine dioxide, benzoic acid, and 1-MCP were limited [38,58,59]. 1-MCP significantly inhibits the transcription of key flavonoid biosynthetic enzymes and ethylene perception proteins, but not the flavonoid transport enzyme [51]. In our present study, we speculated that among different treatments, chlorine dioxide may be more effectively promoted the expression of the key functional differential genes of flavonoid biosynthesis pathways such as *Bra008792*, *Bra009358*, and *Bra027457*, which resulted in delaying flavonoid degradation and further increasing the content of flavonoids. Thus, these findings can provide new insight into the metabolic shifts in postharvest flowering Chinese cabbage during storage.

## 3. Materials and Methods

### 3.1. Materials and Reagents

The variety ‘sijiu’ of flowering Chinese cabbage was used in this study. The fresh postharvest flowering Chinese cabbage samples were collected from Four Seasons Green Farm (Huizhou City, China) on 29 October 2019. Harvested flowering Chinese cabbages were transported to the laboratory under low temperature, and treated with the control, benzoic acid, chlorine dioxide and 1-MCP immediately after arrival in the laboratory on 29 October 2019. These samples were divided into four treatment groups: control, benzoic acid, chlorine dioxide, and 1-MCP. It was determined in a previous pilot experiment that the application of 0.265 g L^−1^ benzoic acid or chlorine dioxide was more effective to delay leaf senescence of Chinese flowering cabbage than treating with 0.1325, 0.53 and 1.06 g L^−1^, and 3 μL L^−1^ 1-MCP was more effective to delay leaf senescence than treating with 1, 2, and 4 μL L^−1^ (Figure S8). Therefore, benzoic acid, chlorine dioxide, and 1-MCP (0.265 g L^−1^, 0.265 g L^−1^, and 3 μL L^−1^, respectively) were selected in the following experiments. Each treatment group contained three replicates and 20 plants per replicate. The control group was sprayed with pure water. The benzoic acid and chlorine dioxide treated groups were sprayed with 0.265 g L^−1^ concentration, respectively, and the spraying dosage was 0.02 L per replicate. After spraying, each replicate sample was put into a polyethylene (PE) bag (40 × 30 cm^2^) and sealed. In the 1-MCP treated group, each replicate sample was packed in a PE bag with 3 μL L^−1^ 1-MCP (0.25 g of dry powder with 0.1 mL water) and sealed. Benzoic acid, chlorine dioxide, and 1-MCP were used only one time for the harvested flowering Chinese cabbages on 29 October 2019 during the experiment. The samples of the four treated groups were stored immediately at 4 °C after treatment, until their phenotype had dramatically changed, and then analyzed for their physiological qualities and transcript-metabolite profiling. Analytical-grade chemical reagents were used in the study (Alfa Aesar, Beijing, China).

### 3.2. Quality Analysis

The samples of the postharvest flowering Chinese cabbage treated with benzoic acid, chlorine dioxide, and 1-MCP were stored at 4 °C. When their phenotype had dramatically changed (stored for 20 d), the weight loss, the concentration of chlorophyll, ascorbic acid, soluble protein, soluble sugar, total polyphenol, and total flavonoids of these postharvest flowering Chinese cabbage were analyzed.

#### 3.2.1. Determination of Chlorophyll

The chlorophyll concentration of the control, benzoic acid, chlorine dioxide, and 1-MCP treatments in the postharvest flowering Chinese cabbage was measured according to the conventional method [60]. The results were expressed on a fresh weight basis as g kg^−1^.

#### 3.2.2. Determination of Ascorbic Acid

The ascorbic acid concentration of the control, benzoic acid, chlorine dioxide, and 1-MCP treatments in the postharvest flowering Chinese cabbage was performed using a modified method [61]. The results were expressed on a fresh weight basis as g kg^−1^.

#### 3.2.3. Determination of Soluble Protein

The soluble protein concentration of the control, benzoic acid, chlorine dioxide, and 1-MCP treatments in the postharvest flowering Chinese cabbage was evaluated based on Bradford’s method, with bovine serum albumin (Sangon Biotech, Shanghai, China) as the standard [62]. The results were expressed on a fresh weight basis as g kg^−1^.

#### 3.2.4. Determination of Soluble Sugar

The soluble sugar concentration of the control, benzoic acid, chlorine dioxide, and 1-MCP treatments in the postharvest flowering Chinese cabbage was evaluated using the Anthrone method [63]. The results were expressed on a fresh weight basis as g kg^−1^.

#### 3.2.5. Determination of Total Polyphenolic

The total polyphenolic concentration of the control, benzoic acid, chlorine dioxide and 1-MCP treatments in the postharvest flowering Chinese cabbage was evaluated based on Folin-Denis reagent, using gallic acid (Sangon Biotech, Shanghai, China) as the standard phenol [64]. The results were expressed on a fresh weight basis as g of gallic acid equivalent (GAE) per kg (g GAE kg^−1^).

#### 3.2.6. Determination of Total Flavonoids

The total flavonoid concentration of the control, benzoic acid, chlorine dioxide, and 1-MCP treatments in the postharvest flowering Chinese cabbage was evaluated using the Zuo method [65]. The results are expressed on a fresh weight basis as g kg^−1^.

### 3.3. Transcriptome-Metabolite Profiling

#### 3.3.1. Transcriptome Sequencing and Analysis

The transcript analysis of postharvest flowering Chinese cabbage treated with chlorine dioxide, benzoic acid, and 1-MCP was performed according to a modified CTAB method [66,67,68]. In brief, a number of different postharvest flowering Chinese cabbages (each vacuum freeze-drying sample, 100 mg, 3 repetitions) were treated with benzoic acid, chlorine dioxide, and 1-MCP, which was applied to construct the transcriptome library and Illumina sequencing (Metware Biotechnology Co., Ltd. Wuhan, China). The Illumina HiSeq™ 2500 platform (Illumina Inc., San Diego, CA, USA) was used for the transcriptome library paired-end sequencing. The RNA concentration was determined on a NanoDrop 2000 spectrophotometer (Thermo Fisher Scientific, Waltham, MA, USA). The RNA integrity was determined on Agilent 2100 system (Agilent Technologies Inc., Palo Alto, CA, USA). The reference genome was used to co-map the Hierarchical Indexing program for Spliced Alignment of Transcripts (HISAT 2) [69]. High-quality clean reads (clean data) were acquired from the mixed RNA samples of different postharvest flowering Chinese cabbage treated with benzoic acid, chlorine dioxide, and 1-MCP to establish the reference transcriptome for further differential analysis.

The differential expression analysis was performed by the software DESeq to obtained DEGs. The Benjamini-Hochberg method was applied to measure the *p*-value. A *p*-value of <0.05 for the DEGs was screened through the DESeq software 1.22.2 (Michae I Love, Chapel Hill, NC, USA). The function of DEGs was annotated according to the methods reported by Rothenberg [66] combined with the public databases: NCBI non-redundant protein sequences (Nr), Kyoto Encyclopedia of Genes and Genomes (KEGG), homologous protein family (Pfam), Gene Ontology (GO), Swiss PROT protein sequence database, and Clusters of Orthologous Groups of proteins (COG/KOG). GOseq R package software was used for the GO analysis of DEGs. The KEGG Orthology Based Annotation System (KOBAS) software 3.0 (Dechao Bu, Beijing, China) was used for the identification of the enriched DEG pathways.

#### 3.3.2. Metabolite Detection and Analysis

Based on the public metabolic database combined with the MetWare database (Metware Biotechnology Co., Ltd. Wuhan, China), metabolites in postharvest flowering Chinese cabbage treated with benzoic acid, chlorine dioxide, and 1-MCP were identified. The metabolite analysis of different postharvest flowering Chinese cabbage (each vacuum freeze-drying sample, 100 mg, 3 repetitions) treated with benzoic acid, chlorine dioxide, and 1-MCP was performed with a standard method [69]. Briefly, the metabolite was normalized by the PCA and OPLS-DA. The variable importance of these obtained data in the project (VIP) ≥1 with a fold change ≥2 or ≤0.5 were defined as DAMs with significant differences in content, and a false discovery rate (FDR) <0.05 was considered as the significant enriched KEGG pathways in Fisher’s exact test. Furthermore, gene-metabolite pairs with a Pearson’s correlation coefficient > 0.8 were used to construct the transcript-metabolite network.

### 3.4. qRT-PCR Analysis of Functional Genes

Based on the result of transcript analysis, the key functional DEGs were confirmed by qRT-PCR. The total RNA was extracted using the Plant RNA extraction kits (RNeasy plant mini kit, Qiagen, Hilden, Germany). In brief, the total RNA of 100 mg sample powder treated with TRIzol™ reagent was extracted (3 repetitions). After that, the purity and concentration of total RNA using cDNA synthesis kits with an oligo-adaptor primer were measured, and then the total RNA was reverse-transcribed into cDNA. The primers (Table S1) used in qRT-PCR are all designed by NCBI online website and synthesized by Beijing Kinco Biological Co., Ltd. The BIO-RAD CFX96 instrument was used to analyze and quantify the qRT-PCR results using relative quantification software. The GAPDH gene was used as an internal reference gene.

### 3.5. Confirmation of Functional Metabolites with HPLC

Based on the result of metabolite analysis, the functional differentially flavonoids were confirmed by HPLC. The functional differentially flavonoids were confirmed in different chromatographic conditions. The chromatographic conditions for catechin, chalcone, taxifolin, and hesperetin were YJYF-009 C18 bar (5 μm, 4.6 mm × 250 mm), with a gradient elution of acetonitrile and 0.1% phosphoric acid aqueous solution, at 25 °C, the detection wavelength at 283 nm. The chromatographic conditions for quercetin-3-O-rutinoside (rutin) were ACQUITY UPLC BEH C18 bar (1.7 μm, 2.1 mm × 50 mm), with a gradient elution of methanol and 0.1% acetic acid aqueous solution, at 30 °C, the detection wavelength at 360 nm. The chromatographic conditions for kaempferol and nicotiflorin were Agilent Poroshell 120 EC-C18 column (2.7 μm, 3.0 mm × 50 mm), with a gradient elution of 0.1% formic acid solution and acetonitrile, at 30 °C, the detection wavelength at 360 nm. The chromatographic conditions for baicalein were Waters Sunfire C18 bar (5 μm, 4.6 mm × 250 mm), with a gradient elution of acetonitrile and 0.1% phosphoric acid aqueous solution, at 30 °C, the detection wavelength at 360 nm. The HPLC results were expressed on a fresh weight basis as g kg^−1^.

### 3.6. Statistical Analysis

The measurements in the present experiments were performed in triplicate. The data was expressed using mean ± standard deviations (SD). The statistical analyses were measured using ANOVA in the software SPSS v20.0 (IBM, Armonk, NY, USA). Build of the PCA models were performed by using R language v1.3.959 (R Core Team, Vienna, Austria). The *p*-value of <0.05 or 0.01 was considered as statistically significant.

## 4. Conclusions

Herein, we evaluated the effects of benzoic acid, chlorine dioxide, and 1-MCP on the physiological qualities and the metabolic shifts in postharvest flowering Chinese cabbage during storage. Benzoic acid, chlorine dioxide, and 1-MCP could delay the senescence and nutrient degradation, and the effect of 1-MCP was the best. Furthermore, benzoic acid, chlorine dioxide, and 1-MCP had different effects on the DEGs and DAMs annotated to the flavonoid biosynthesis pathways, and different treatments could delay or promote the degradation of flavonoids by regulating the expression of different related genes. The effects of the treatments on each kind of flavonoid and the same treatment on different flavonoids were different. Interestingly, the analysis of the transcript-metabolite correlation network revealed that although 1-MCP showed the best effect of prolonging the shelf life among the treatments, chlorine dioxide could simultaneously promote the expression of the four key differential genes (*Bra007142*, *Bra008792*, *Bra009358*, and *Bra027457*) that were confirmed using qRT-PCR technology. These key differential genes played a pivotal role in delaying the degradation of flavonoids (hesperetin, chalcone, rutin, and baicalein). Thus, our present findings will contribute to a better understanding of the comparative and scientific effects of benzoic acid, chlorine dioxide, and 1-MCP on the preservation of flowering Chinese cabbage during storage.

## Data Availability

Not applicable.

## Supplementary Materials

The following supporting information can be downloaded at: https://www.mdpi.com/article/10.3390/ijms23116011/s1.
