# Supplementary material for: Benzoic Acid, Chlorine Dioxide, and 1-Methylcyclopropene Induce Flavonoid Metabolic Shifts in Postharvest Flowering Chinese Cabbage Revealed by High-Dimensional Analytical Data
Source: Int J Mol Sci. 2022 May 27;23(11):6011. doi: 10.3390/ijms23116011 (PMC9180784; doi:10.3390/ijms23116011)
Supplement: Supplementary file 1 [file ijms-23-06011-s001.zip › ijms-1730424-supplementary.pdf]

1. Tables:

Table S1. Primer sequence

Primer name	Primer sequence (5'-3')
GAPDH-F	GCTAACTGCCTTGCTCCACTT
GAPDH-R	CGGCTCTTCCACCTCTCCAG
Bra008792-F	ATGGTGATGGGTACACCGTC
Bra008792-R	TGTCGCACATGCGCTTGAAC
Bra009358-F	GGTATAAGAATGTGTTGCAT
Bra009358-R	TCAGTCCAGAGGAAGCTTAT
Bra027457-F	GTCAAAAGGGTTTTCTCCCT
Bra027457-R	CTAAGCACAGATCTGCTGTG
Bra007142-F	TTGAAAACAAGTTGTTGGCA
Bra007142-R	TCAGTTCTCTTTGGCCAGTT

Table S2. The metabolites of BA vs. CD groups

Index	Compounds	VIP	Fold_Change	LogFC	Type
pme0241	Benzoic acid	2.33	6.40	2.68	up
mws0103	Indole-3-carboxaldehyde	1.38	2.02	1.01	up
pmp001291	Citropten	1.33	2.01	1.05	up
Hmcp003852	Elemol	1.29	0.50	1.01	down
Lmhn003240	Benzoylmalic acid	3.14	25.75	4.69	up
mws0054	Catechin	4.93	0.01	11.52	down
mws1033	Homoeriodictyol	3.45	0.21	2.24	down
mws0044	Taxifolin	1.45	2.22	1.15	up
pmp000107	Monohydroxy-trimethoxyflavone	1.50	2.28	1.19	up
pmf0557	Shikonin	1.13	2.15	1.10	up
pmn001695	Trihydroxycinnamoylquinic acid	2.53	8.30	3.05	up
Hmxn000778	2(R)-Hydroxy-3-butenyl glucosinolate	1.36	0.50	1.01	down

Cmln001085	2-Hydroxy-2-Methylbutyl Glucosinolate	1.50	0.40	1.34	down
CMLN001809	3-Methylsulfinylpropyl glucosinolate	1.54	0.38	1.40	down
Lmyn003825	4-Methylsufinyl-3-Butenyl Glucosinolate	3.79	4.57	2.19	up
CMLN001810	5-Methylthiopentyl Glucosinolate	2.13	0.20	2.31	down
pmn001409	Plantainoside A	1.70	0.37	1.44	down
Zmhn003565	Kaempferol-3-O-(6"-acetyl)-glucoside	2.06	0.20	2.35	down
pmb3026	Quercetin-O-acetylhexoside	1.67	0.37	1.46	down
pmb3041	Tricin O-saccharic acid	1.60	0.41	1.30	down
pmb0746	Tricin 4'-O-β-guaiacylglycerol	1.31	2.02	1.01	up
Hmln002483	Kaempferol-3-O-(6"-malonyl)-glucoside	1.95	0.23	2.11	down
pme0368	Apigenin 7-rutinoside(Isorhoifolin)	2.49	0.01	18.21	down
pme3558	6,8-di-C-glucoside apigenin	2.26	0.01	14.63	down
pmp000593	Luteolin-7-O-rutinoside	1.28	0.12	3.01	down
GQ512005	Kaempferol-3-O-glucoside-7-O-rhamnoside	1.21	0.12	3.03	down
mws1073	Apigenin 6,8-C-diglucoside	2.24	0.00	14.41	down
pme0369	Kaempferol-3-O-rutinoside(Nicotiflorin)	1.35	0.08	3.65	down
pmp001080	Neodiosmin (Diosmetin-7-O-Neohesperidoside)	1.65	0.13	2.99	down
mws1661	Diosmin	1.52	0.14	2.80	down
Hmmp002336	Quercetin-O-feruloyl-Pentoside	1.65	0.04	4.66	down
mws0059	Quercetin-3-O-rutinoside (Rutin)	2.25	0.01	14.45	down
pmb0711	Quercetin-7-O-rutinoside	1.65	0.04	4.52	down
pmn001583	Quercetin-3-O-robinobioside	3.80	0.02	5.93	down
Hmqp003184	Luteolin-O-Malonyl-O-Hexoside-O-Pentoside	3.83	0.18	2.46	down
pmb0709	Quercetin-7-O-malonylhexosyl-hexoside	1.46	0.45	1.16	down

2. Figures:

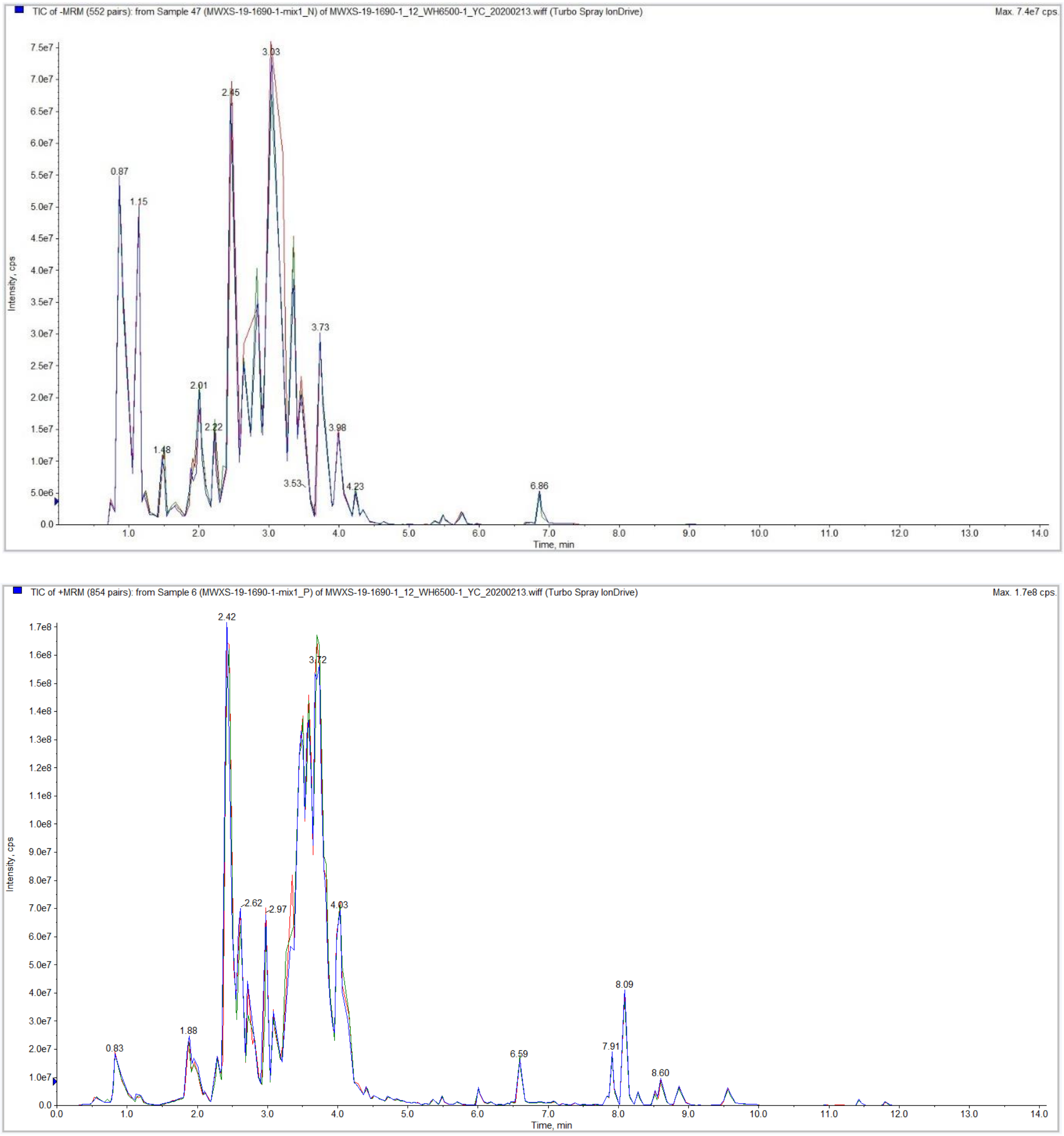

Figure S1: The total ion flow diagram was detected by the essential spectrum of quality control samples

A

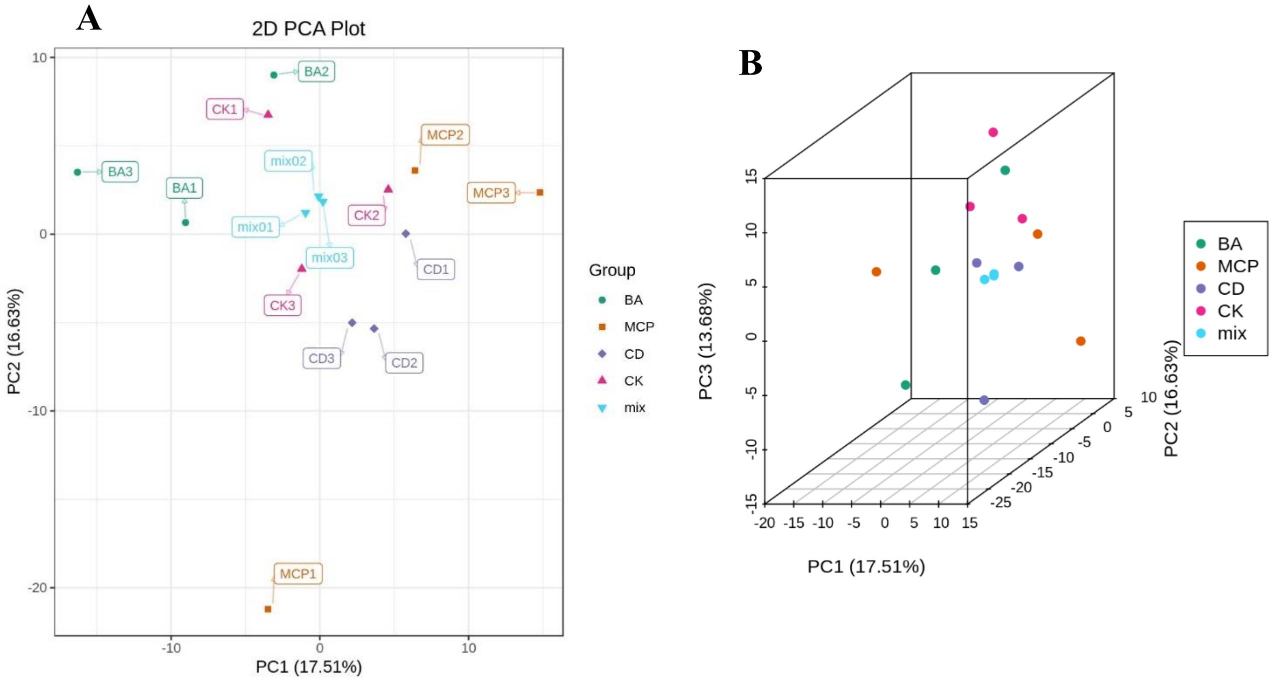

Figure S2: PCA score chart of quality spectrum data of each group of samples and quality control samples (A,B)

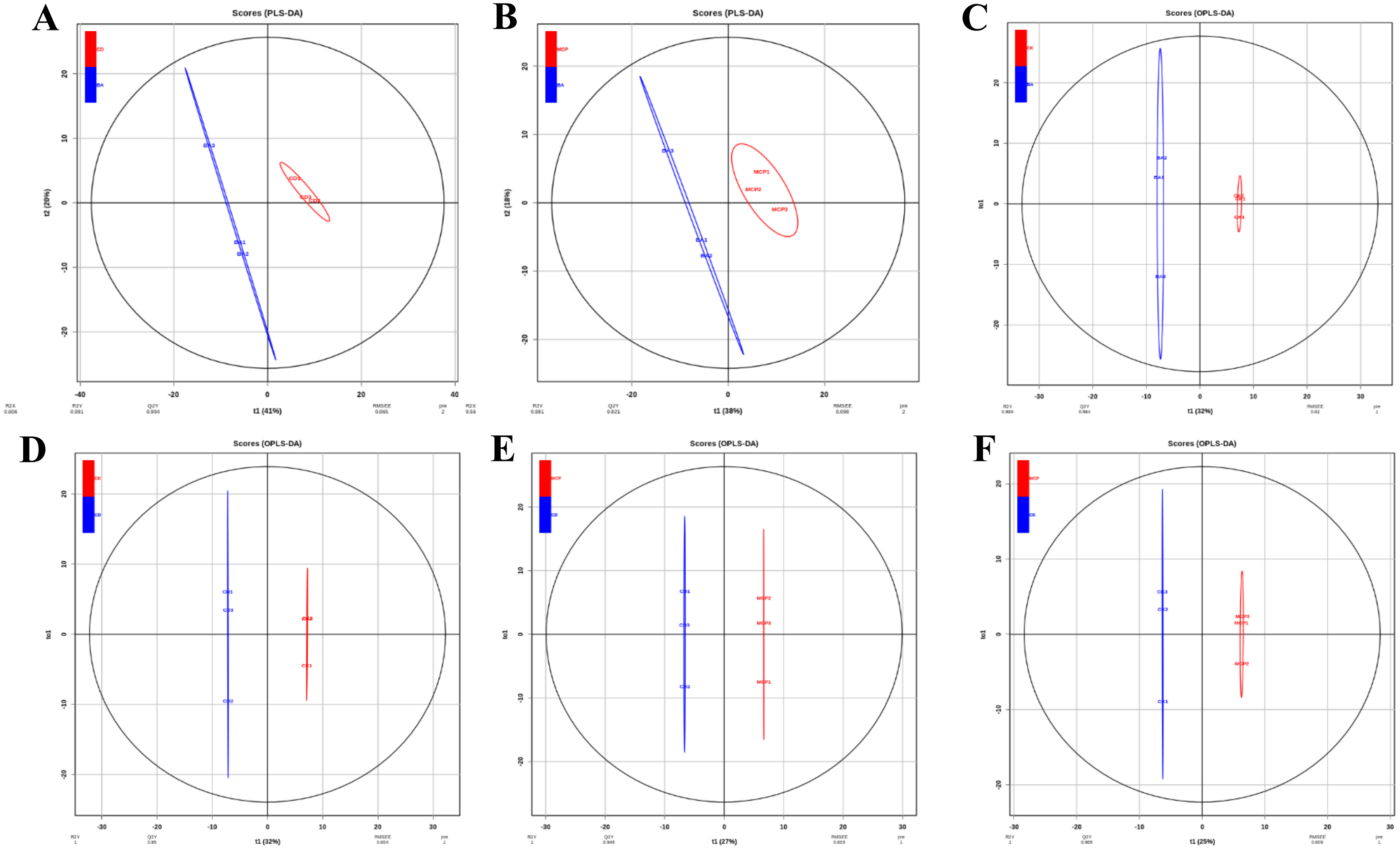

Figure S3: Orthogonal partial least square discriminant analysis (OPLS-DA) score graph in different combination of BA vs CD (A), BA vs MCP (B), BA vs CK (C), CD vs CK (D), MCP vs CD (E), MCP vs CK (F).

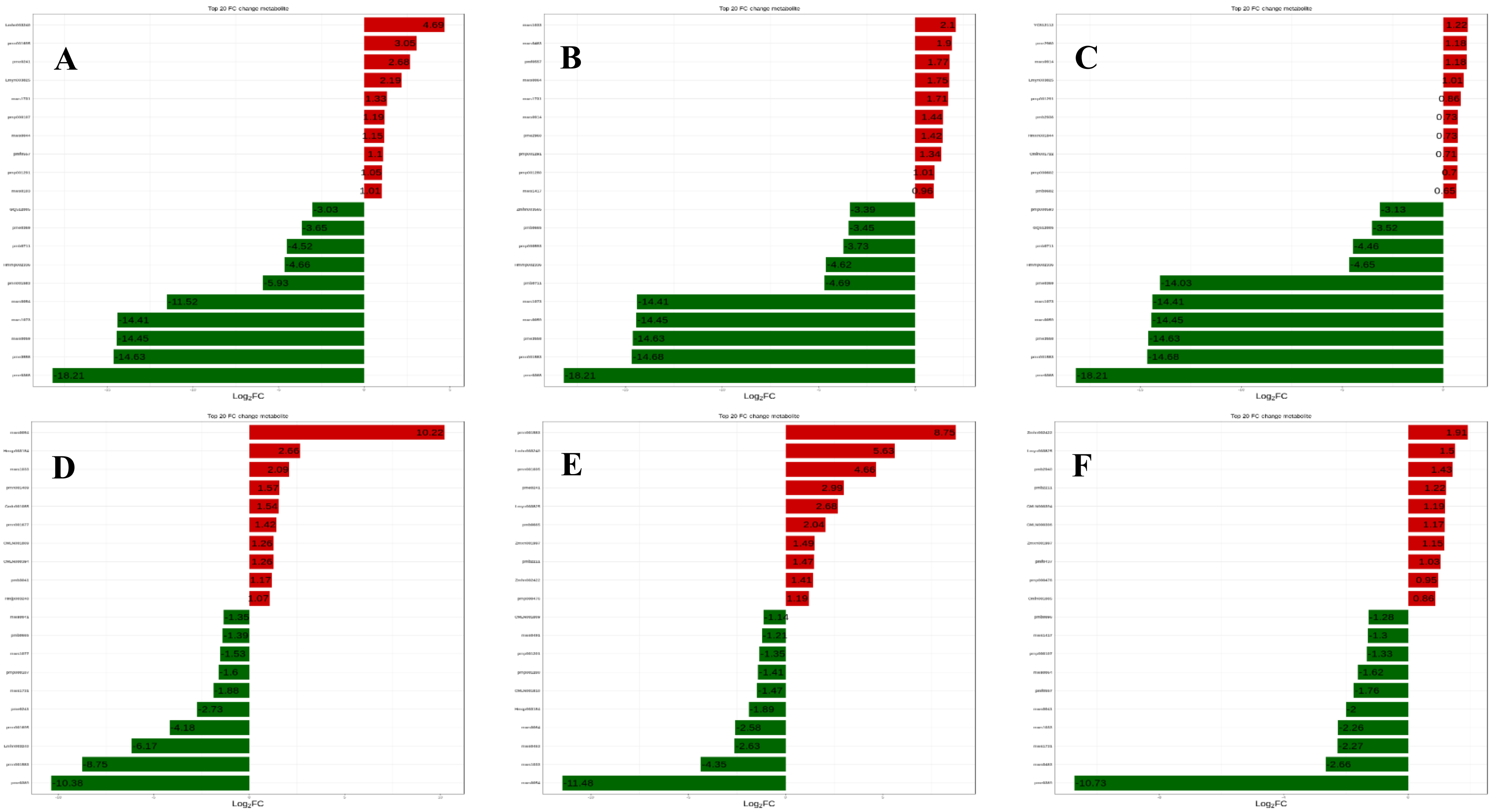

Figure S4: Top 20 FC change metabolite in different combination of BA vs CD (A), BA vs MCP (B), BA vs CK (C), CD vs CK (D), MCP vs CD (E), MCP vs CK (F).

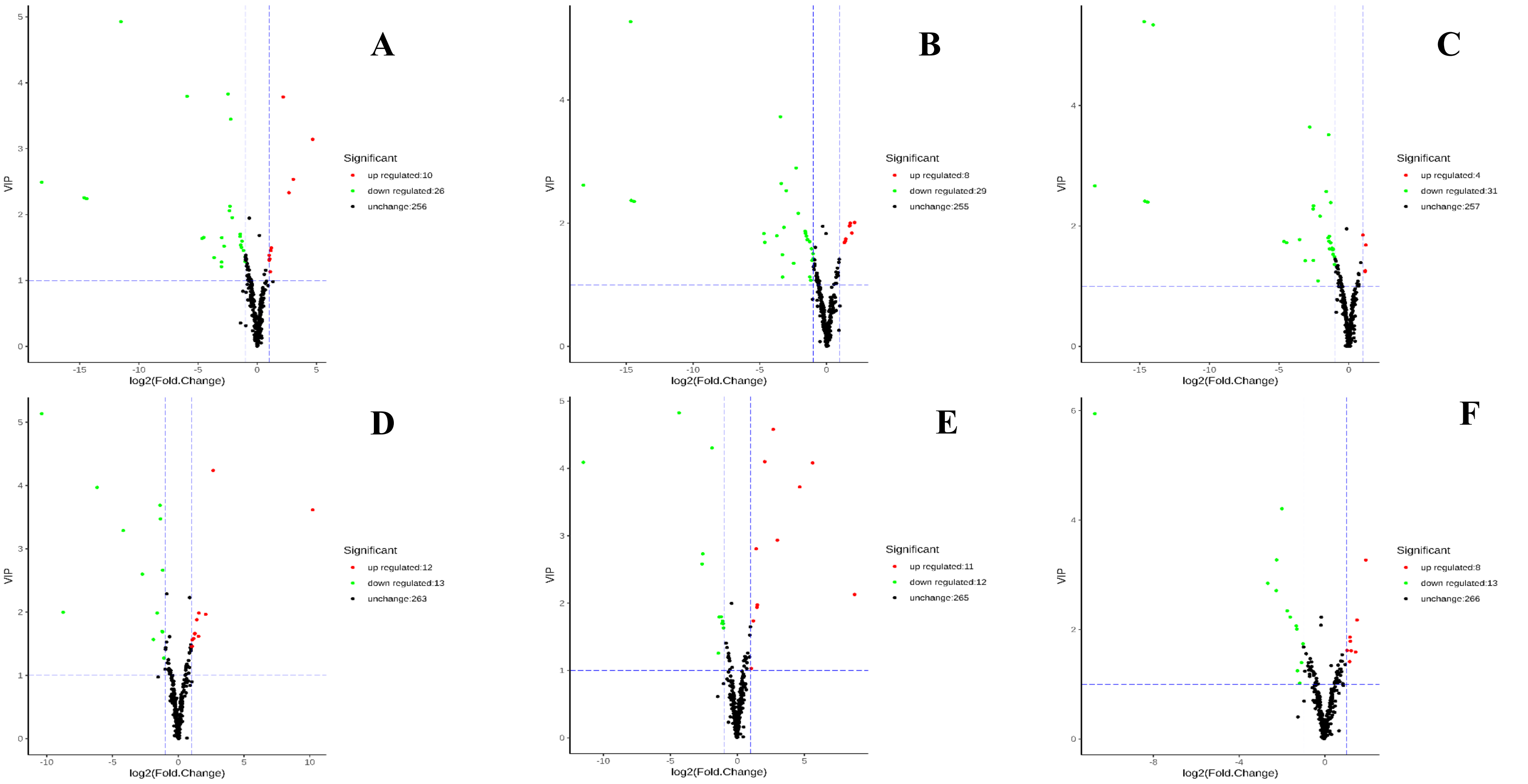

Figure S5: Volcano map of differential metabolite in different combinations of BA vs CD (A), BA vs MCP (B), BA vs CK (C), CD vs CK (D), MCP vs CD (E), MCP vs CK (F).

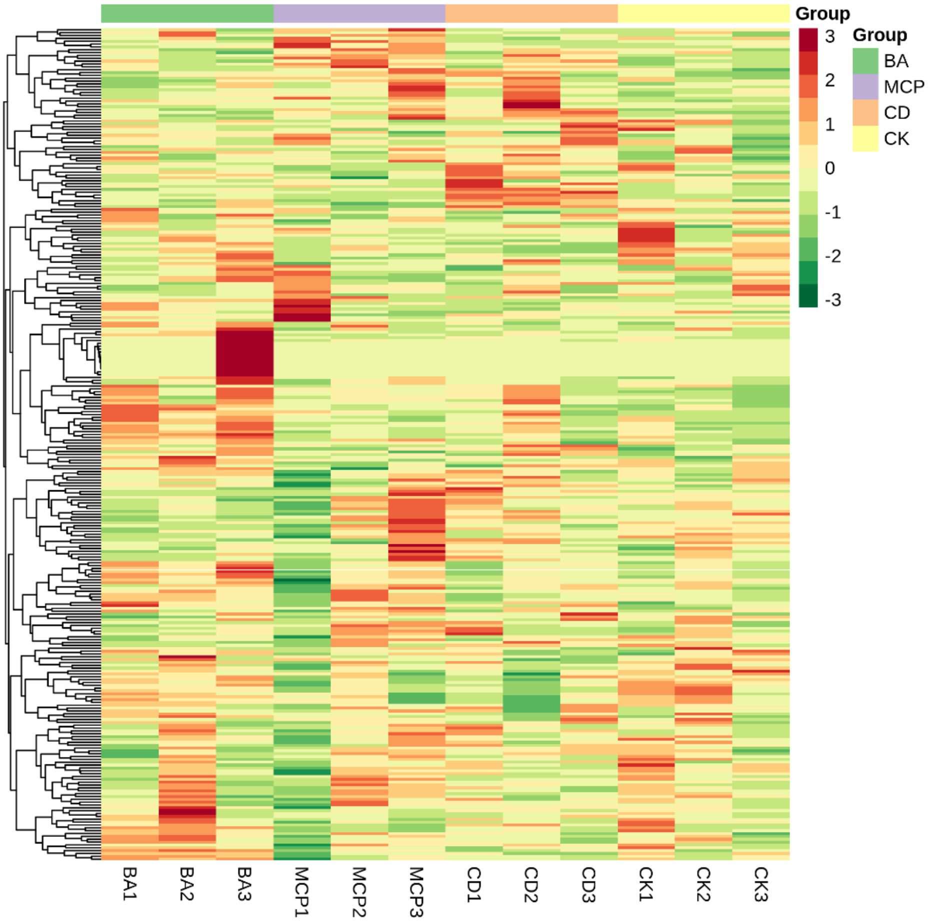

Figure S6: clustering heatmap of differential metabolite

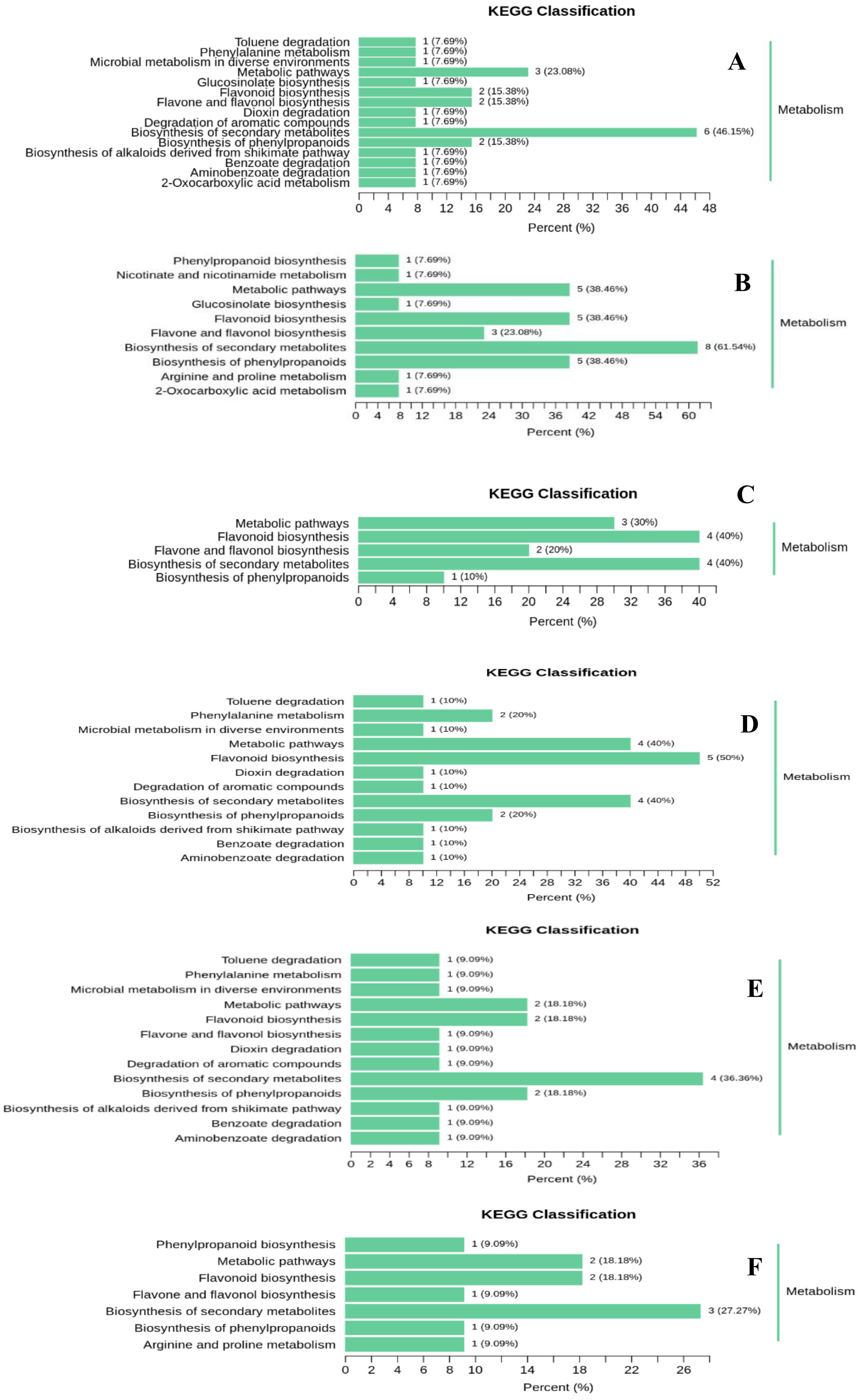

Figure S7: KEGG classification of differential metabolite in different combinations of BA vs CD (A), BA vs MCP (B), BA vs CK (C), CD vs CK (D), MCP vs CD (E), MCP vs CK (F).

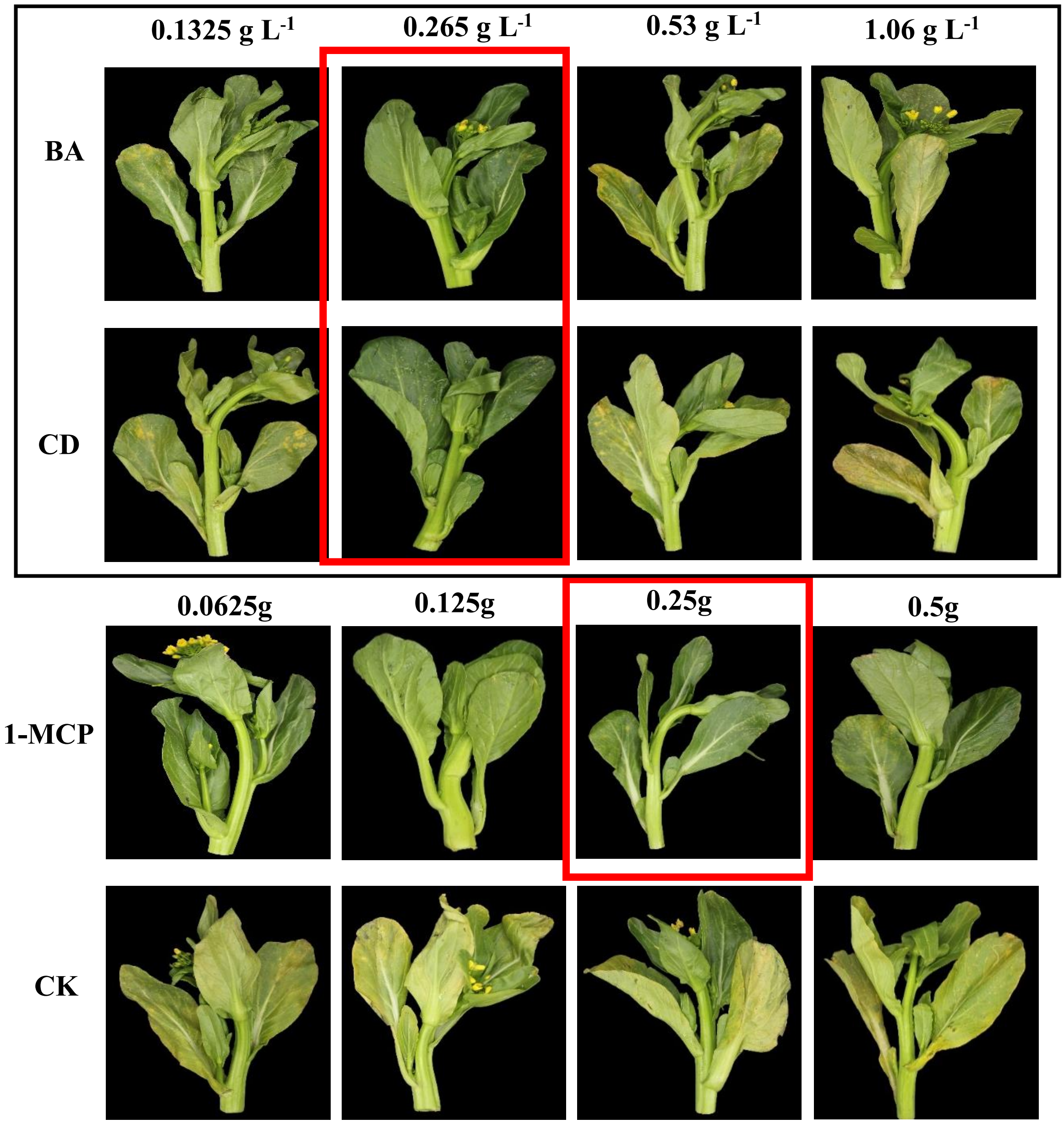

Figure S8: The effects of different concentrations of benzoic acid, chlorine dioxide and 1-MCP on the phenotype of flowering Chinese cabbage at 4 °C for storage 20 days.
